# The physiological and psychological relaxing effects of viewing rose flowers in office workers

**DOI:** 10.1186/1880-6805-33-6

**Published:** 2014-03-08

**Authors:** Harumi Ikei, Misako Komatsu, Chorong Song, Eri Himoro, Yoshifumi Miyazaki

**Affiliations:** 1 Center for Environment, Health and Field Sciences, Chiba University, Chiba, Japan; 2 Mizuho Information & Research Institute, Inc, Tokyo, Japan

**Keywords:** Fresh rose flowers, Office workers, Visual stimuli, Heart rate variability, Pulse rate

## Abstract

**Background:**

In recent years, the physiological relaxing effect brought by nature is becoming clear; however, many workers find it difficult to be exposed to nature in their working environment. Exposure to fresh flowers represents an opportunity to incorporate nature into their working lives. In this study, we examined the effects of exposure to roses on physiological and psychological variables (heart rate variability, pulse rate, and subjective responses) in office workers.

**Results:**

The experimental site was Mizuho Information & Research Institute, Inc., in the Tokyo metropolitan area. Thirty-one male office workers were included in the present study. The subjects were exposed to thirty unscented pink roses (Rosa, Dekora) arranged in a cylindrical glass vase for 4 min. In the control condition, the subjects were not exposed to flowers. After the experiments, the subjects completed a questionnaire. The order of exposure was counterbalanced among subjects. Among subjects exposed to roses, the high-frequency component of heart rate variability was significantly higher than in controls. Similarly, 'comfortable,’ 'relaxed’ and 'natural’ feelings were more common in subjects exposed to roses.

**Conclusions:**

Data from this study support the presence of physiological and psychological relaxing effects of being exposed to flowers on office workers.

## Background

Ever increasing urbanization and job pressures have resulted in an overly stressed society far removed from potential calming effects of nature [1]. The disconnect between this state of permanent urban stress and our evolutionary history in the natural world was encapsulated by Miyazaki *et al*. [1]: "We have become the humans we are today, living in a modern civilization, through a process of evolution that took place in a natural environment. The human body is thus made to adapt to nature. However, as conveyed through terms such as ‘techno-stress’, artificialisation is taking place at such a rapid rate that we now find ourselves in stressful situations in our daily lives and are forced to deal with the resultant pressures [1]". We hypothesise that modern people in stressful states can be relaxed through contact with nature.

In our stressful modern societies, the relaxing effect of natural stimuli is considered advantageous compared with other stimuli. Many people are thus attracted to the physiological and psychological relaxing effect of exposure to nature. Field experiments on forest bathing [2-10], urban parks [11] and rooftop gardens [12] have demonstrated physiological relaxing effects of contact with nature. Furthermore, Li *et al*. reported forest bathing increased natural killer cell function and improved immune function [13]. This effect was sustained for approximately 1 month. The results of these studies suggest that contact with nature is a type of prophylaxis.

Globally, evidence-based medicine has been attracting attention, with physiological data from field tests offering great value. We expect that accumulating physiological data from field experiments will continue to demonstrate preventive medical effects of nature therapy in the future [1,14].

In modern society, many people spend the majority of their time in intensely stressful states, and have no time for contact with nature outside of their immediate surroundings. Office workers who work in urban areas are typical examples. Previous studies have evaluated the psychological stress levels among office workers [15], and they have shown that stressors at work not only cause psychological symptoms but also increase risk of cardiovascular disease [16] and conditions such as insomnia [17]. Moreover, data reported by the National Police Agency indicate that work problems were responsible for 8.2% cases of suicide in Japan in 2010 [18]. Therefore, because many office workers work in highly stressful environments, it might be argued that alleviating this situation is matter of urgency. However, most previous studies [2-10] had male college students as subjects, and there are few studies of office workers aged 20 to 50 years.

Flower arrangements are familiar to many people as bringing nature into daily life and offer office workers easy contact with nature within the constraints of time and space. In the Japanese flower market, chrysanthemum is the most popular flower type, with the most popular flowers being carnations and roses [19]. Because chrysanthemums are usually laid for the deceased, roses are one of the most preferred flowers in Japan. The relaxing effects of fresh flowers such as roses are empirically known. There are many studies of psychological effects of exposure to flowers, but none have objectively studied the physiological effects of such exposure. We propose that flower arrangements may contribute to improving the stress in daily life from the viewpoint of preventive medicine. The aim of this research is to clarify the physiological and psychological relaxing effects of exposure to rose flowers on male office workers experiencing high levels of stress.

## Methods

The experiments were conducted in a conference room of the Mizuho Information & Research Institute, Inc. The room temperature was maintained at 24.6°C ± 1.8°C (control room, 25.2°C ± 0.7°C) and relative humidity was maintained at 31.6% ± 3.9% (control room, 29.7% ± 1.8%). Thirty-one male workers (37.3 ± 2.0 years old) working in the institute were included. The study was conducted with the approval of the Ethics Committee of the Center for Environment, Health and Field Sciences, Chiba University and all subjects provided written informed consent.

**Figure 1:**
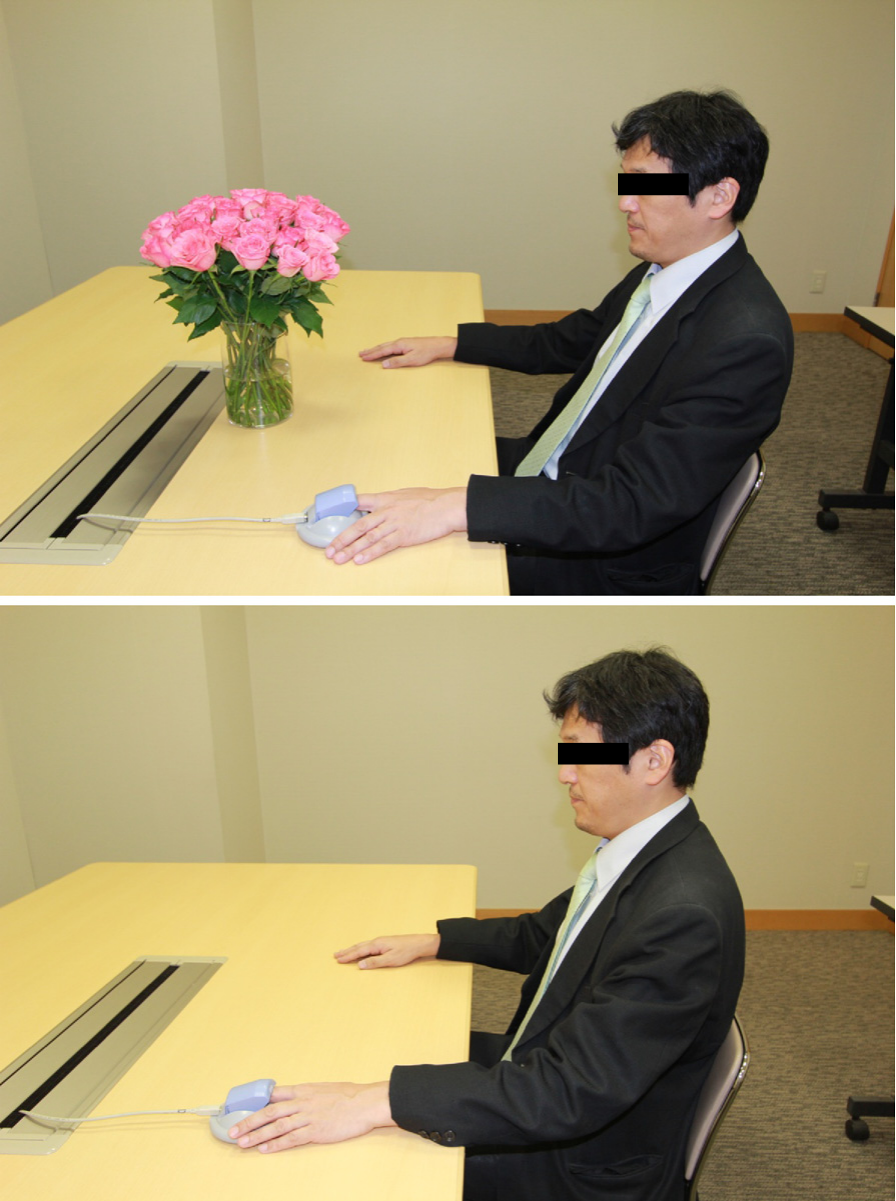
**Experimental setting.** The physiological indices of a subject sitting in the viewing area of the roses and control are being measured.

Thirty fresh and unscented (sensory intensity was classed as unscented on testing) pink roses (*Rosa*, cultivar name: Dekora) trimmed to a length of 40 cm were used for viewing. The roses were arranged in a cylindrical glass vase 12 cm in diameter and 20 cm in height. The distance from subjects’ eyes to the flowers was 37 to 40 cm, and it was adjusted according to the height of the subjects. The experimental setting is shown in Figure 1.

We explained the experimental protocol to subjects in the waiting room, after which they received 4 minutes of viewing roses. The control group was not exposed to flowers in another room. After the experiments, subjects filled a questionnaire. The order of stimuli was counterbalanced among subjects.

**Figure 2:**
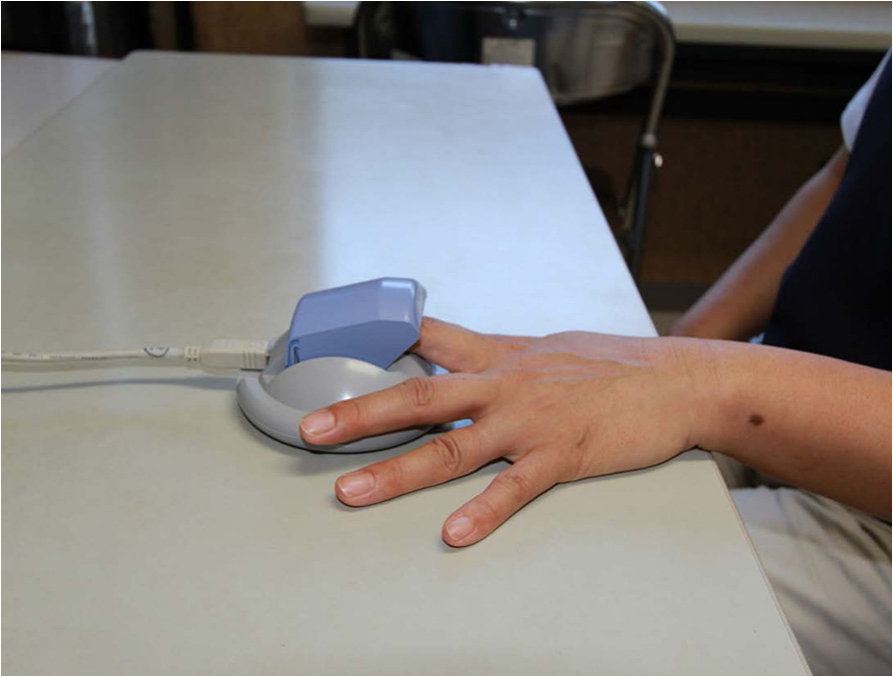
An example of the fingertip pulse wave.

Previous studies have reported that the a-a intervals of the acceleration plethysmograph (APG) and the R-R intervals of electrocardiographs are highly correlated [20]. Therefore, heart rate variability (HRV) was calculated by spectral analysis of the coefficient of the variation of the a-a intervals of an APG shown in Figure 2 (ARTETT, U-Medica Inc., Osaka, Japan). The pulse rate was converted by dividing 60 with a-a intervals of APG. The sampling frequency was set at 1,000 Hz. The maximum entropy method was used for frequency analysis, and variance of the low-frequency (LF) band (0.04 to 0.15 Hz) and high-frequency (HF) band (0.15 to 0.40 Hz) were calculated. The LF/HF ratio for R-R interval variability was also assessed. The HF component was used as an index of parasympathetic nervous activity and the LF/HF ratio was as an index of sympathetic nervous activity [21]. The HRV and pulse rate data were collected continuously during the 4 minutes of the experiments and an average was calculated over 4 minutes.

For psychological evaluations, we employed a 13-point rating scale including the following parameters: ‘comfortable-uncomfortable’, ‘relaxed-awakening’ and ‘natural-artificial’ along with the factors in the profile of mood states (POMS) questionnaire. The POMS questionnaire simultaneously evaluates six measures of mood: tension-anxiety (T-A), depression-dejection (D), anger-hostility (A-H), fatigue (F), confusion (C), and vigor (V). In this study, were used the Japanese version of POMS [22-24] consisting of 30 items.

The Statistical Package for Social Sciences software (v20.0, SPSS Inc., Chicago, IL, USA) was used for all statistical analyses. Statistical analysis of physiological data was performed using the paired *t*-test after calculating the average values obtained during the 4-minute viewing of the roses versus those obtained during the control condition. Psychological evaluation was analyzed by the Wilcoxon signed-rank test. Statistical differences were considered significant at *P* <0.05.

## Results and discussion

**Figure 3:**
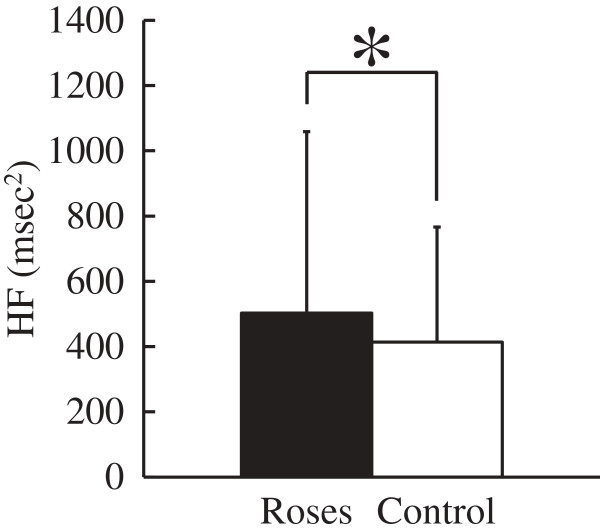
**Comparison of the mean values of the high-frequency (HF) component between the rose flower exposure and control condition.** N = 31; mean ± SD; *P <0.05; significant differences verified by paired *t*-test.

Figure 3 shows the HF component of HRV. The HF component was 502.4 msec^2^ during rose flower viewing, and 414.0 msec^2^ in the control condition; it showed 21% significant increase (*p* <0.05) on exposure to roses, meaning that parasympathetic nervous activity was significantly higher while viewing the roses. There were no significant differences in LF/HF of HRV or pulse rate.

**Figure 4:**
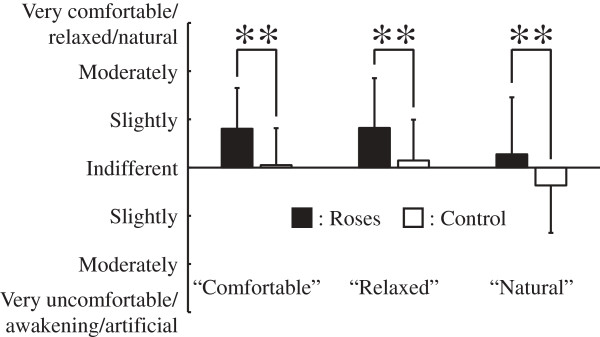
**Changes in subjective evaluation for ‘comfortable-uncomfortable’, ‘relaxed-awakening’ and ‘natural-artificial’ with the rose flower exposure and control conditions.** N = 31; mean ± SD; **P <0.01; Wilcoxon signed-rank test.

**Figure 5:**
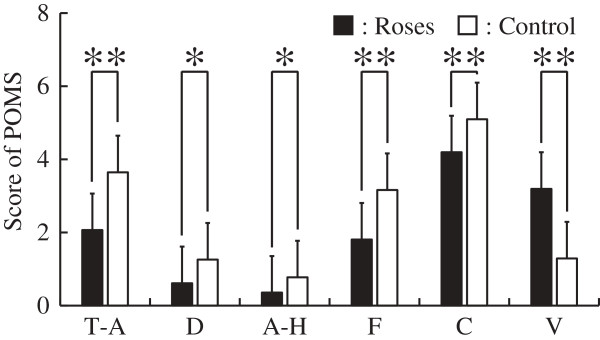
**Subscale scores for the profile of mood states (POMS) scale during the rose flower exposure and control conditions.** N = 31; mean ± SD; **P <0.01; *P <0.05; Wilcoxon signed-rank test. A-H, anger-hostility; C, confusion; D, depression-dejection; F, fatigue; T-A, tension-anxiety; V, vigor.

Figure 4 shows results of comfortable, relaxed and natural states in the subjective evaluation by the questionnaires. Subjects reported being significantly more comfortable being exposed to roses than in the control condition (*P* <0.01). Relaxed and natural states were also significantly more frequent on exposure to roses than in the control condition (*P* <0.01). Figure 5 shows results of the POMS questionnaire survey. Scores of T-A, F and C (*P* <0.01) as well as those of D and A-H (*P* <0.05) were significantly lower on viewing roses than in the control condition. On the other hand, V scores during the rose viewing were significantly higher than in the control condition (*P* <0.01).

In the present study, we evaluated changes in autonomic nervous system activity, a physiological measure of stress, in office workers while viewing common fresh roses [19]. A relatively brief (4-minute) viewing session resulted in significantly increased parasympathetic nervous activity, concordant with several previous studies demonstrating enhanced parasympathetic activity while viewing a forest scene [2,3,6,7,9]. Furthermore, this result is consistent with our previous report demonstrating calming effects of roses in middle-aged and elderly medical staff [25] and in high-school students [26].

Numerous studies have evaluated effects of contact with familiar natural environments on human physiology and emotions. Indoor plants and the view from the window are good examples of the contact with nature obtainable while inside a room, and positive results of their physiological and psychological effects have been demonstrated [27-31]. In the previous studies, it is reported that natural views from hospital rooms or indoor plants hasten recovery of patients after surgery and decrease systolic blood pressure [27-29]. Those effects in an office location have been studied, showing greater work efficiency and job satisfaction simply by placing indoor plants in the office [30,31]. Furthermore, the higher the stress state, the more effective the nature therapy [32].

Our research showed that parasympathetic nervous activity was enhanced by viewing roses. Therefore, by simple stimulus, roses possibly offer preventive medical effects [14] by decreasing stress. Elucidation of the interaction of humans with the natural environment is an important issue in physiological anthropology. The results of this study may have important implications in the work place and for the health of office workers.

This study had a few limitations. First, only male subjects were included, and only roses were viewed. In future experiments, we will examine the responses of female subjects exposed to multiple types of flowers. We predict that the physiological data will support the physiological and psychological relaxing effects of exposure to roses, which may consequently lead to the increased use of flowers to reduce office-related stress. Second, we only evaluated HRV; therefore, this study cannot be considered to be a complete physiological evaluation. Other experimental indices, such as brain activity and stress hormone levels, should be assessed to determine the effects of visual stimulation such as viewing roses on responses in humans.

## Conclusions

Parasympathetic nervous activity was enhanced by viewing roses. This finding suggests a simple method for decreasing stress and improving the health of office workers.

## Abbreviations

A-H: anger-hostility; APG: acceleration plethysmograph; C: confusion; D: depression-dejection; F: fatigue; HF: high frequency; HRV: heart rate variability; LF: low frequency; POMS: profile of mood states; T-A: tension-anxiety; V: vigor.

## Competing interests

The authors declare that they have no competing interests.

## Authors’ contributions

HI contributed to the experimental design, data acquisition, statistical analysis, interpretation of the results and manuscript preparation. MK and CS contributed to acquisition and interpretation of data. EH designed the study and participated in data acquisition. YM had an important role in this research, particularly in study design, interpretation of data and manuscript preparation. All authors contributed to manuscript preparation and are responsible for the final editing and approval of the manuscript.
